# THEY’VE GOT MY BACK: THE ROLE OF PAIN INTENSITY AS A MEDIATOR BETWEEN PERCEIVED RELATIONSHIP QUALITY AND RESILIENCE

**DOI:** 10.1093/geroni/igae098.0207

**Published:** 2024-12-31

**Authors:** Erick Carranza, Landon Peeples, Michael Buxton, M Lindsey Jacobs, Rebecca Allen

**Affiliations:** University of Alabama, Tuscaloosa, Alabama, United States; University of Alabama, Tuscaloosa, Alabama, United States; University of Alabama, Tuscaloosa, Alabama, United States; The University of Alabama, Tuscaloosa, Alabama, United States; University of Alabama, Tuscaloosa, Alabama, United States

## Abstract

Recent increases in the population of aging adults reflect a dire need to examine the social contexts that influence their experiences in relation to physical and mental health, especially during the ongoing coronavirus pandemic. Previous work identifies how perceived care partner relationships play a role in reducing psychological distress for aging adults facing a chronic illness (Schroepfer, 2008). Evidence also suggests that perceived relationship quality is associated with experiencing greater pain and disability (Campbell, 2012). However, little is known about the dynamic interplay between perceived relationship quality, pain intensity, and resilience. This presentation aims to draw upon the Meikirch model of health examining pain intensity as a biologically given potential and relationship quality as personally acquired potential while experiences during COVID-19 may be interpreted as environmental determinants of health. The objective is to understand how relationship quality influences levels of resilience (i.e., psychological well-being) and is then mediated by the intensity of pain. The mediation analysis was conducted using a secondary data approach with responses (N = 2686) from the 2020 wave of the Health and Retirement Study, a nationally representative sample of older adults in the United States. Results revealed a significant indirect effect, indicating that the association between perceived spousal support and resilience decreased when controlling for the intensity of pain experienced by the respondent. Future directions include examining responses from spouses to compare dyadically the perceptions of relationship quality and how these perceptions relate to pain intensity within one partner and dyadic perceptions of couple resilience.

